# Rheumatoid arthritis in crisis: investigating the impact of stress on RA flares during the COVID-19 pandemic

**DOI:** 10.1007/s10067-025-07754-9

**Published:** 2026-01-31

**Authors:** Nicolette T. Morris, Thasia Woodworth, Angela Pham, David A. Elashoff, Jenny Brook, Daniel E. Furst, Veena K. Ranganath

**Affiliations:** 1 Division of Rheumatology, David Geffen School of Medicine, University of California, Los Angeles, Los Angeles, CA USA; 2 The University of Queensland School of Medicine, Ochsner Clinical School, New Orleans, LA USA; 3 University of Texas Southwestern Medical School, Dallas, TX USA; 4 Department of Medicine Statistics Core, David Geffen School of Medicine, University of California, Los Angeles, Los Angeles, CA USA; 5 Division of Rheumatology, University of Washington, Seattle, WA USA; 6 University of Florence, Florence, Italy

**Keywords:** COVID-19, Disease activity, Pandemic, Rheumatoid arthritis, Rheumatoid arthritis flare, Stress

## Abstract

**Introduction/objectives:**

Psychological stress impacts rheumatoid arthritis (RA) disease activity, and California’s response to the COVID-19 pandemic created historically significant stressors for patients. This study examined factors associated with changes in RA flares during the pandemic.

**Methods:**

In this cross-sectional COVID-19 RA study, patients with RA ICD-9/10 codes were emailed a questionnaire in July/November of 2020 containing questions on RA disease activity, Routine Assessment of Patient Index Data 3 (RAPID3), flare number and frequency, RA Flare Questionnaire (RA-FQ), Perceived Stress Scale 4 (PSS-4), stressors, and demographics. Age, anti-cyclic citrullinated antibody, and rheumatoid factor were extracted from medical records. Analyses examined associations between current flare status, number of flares, and changes in flare frequency with PSS-4 and stressors.

**Results:**

Of 1138 respondents (22.6% response rate), 69.3% reported at least one RA flare, 43% multiple flares, and 36.3% currently experiencing a flare. Compared to pre-pandemic levels, 36.3% noted more frequent flares, while 9.2% reported fewer. Increased stress was noted across all flare groups. Regression analyses revealed significant associations between current flare and PSS-4 scores, financial stress, and sleep quality (all *p* < 0.03). A higher number of flares were significantly associated with PSS-4, financial stress, and home stress (*p* < 0.03). Increased flare frequency was associated with PSS-4, apprehension, panic, financial stress, and sleep quality (all *p* < 0.05). Asian race was negatively associated with the number of flares and flare frequency (both *p* < 0.05).

**Conclusions:**

This study reports a link between stress and RA flares during the pandemic, underscoring the need for targeted strategies to manage RA patients at risk of flare during heightened stress.

**Table:** 

**Key Points** • *During the COVID-19 pandemic, patient-reported RA flares increased significantly compared to pre-pandemic levels, signifying there may be value in reevaluating RA management strategies during periods of heightened stress.* • *Financial stress, home stress, and poor sleep were major pandemic stressors linked to current and increased frequency of RA flares.* •* RA patients not in remission may be more susceptible to disease exacerbations while under stress, as those experiencing flares during the pandemic were less likely to have been in remission beforehand.* • *Asian race was associated with fewer RA flares and reduced flare frequency, suggesting racial and ethnic differences may influence flare patterns.*

## Introduction

Rheumatoid arthritis (RA) is a chronic autoimmune disease characterized by relapsing and remitting flares of inflammation [1]. Qualitative evidence indicates that patients often use the term “flare” to describe episodes of significantly worsening RA symptoms, such as joint pain, swelling, and stiffness, that deviate from their usual level of disease activity and consequently impact their quality of life [2, 3]. It is well documented that psychological stress and mood disturbances are common among RA patients and many self-report stress as a key contributor in the course of their disease, flares included [4–7]. The European League Against Rheumatism (EULAR) has recognized the link between psychological distress and RA disease activity in its clinical recommendations, which include stress management as a component for optimizing RA care [8]. However, studying the impact of stress on RA disease activity and flares is challenging due to the wide variability in individual stressors and the difficulties of conducting such studies in large populations.

As one of the most significant global health crises in history, the COVID-19 pandemic caused unprecedented levels of heightened and sustained psychological distress through its disruption of health systems, economies, and societal norms [9]. For RA patients, the pandemic had a profound psychological impact, compounded by unique stressors including concerns about increased infection risk and worse COVID-19 outcomes related to immune dysfunction and use of immunosuppressive medications [10, 11]. In the initial stages of the pandemic, several RA medications, such as hydroxychloroquine and interleukin-6 inhibitors (tocilizumab and sarilumab), were considered potential treatments for COVID-19. This, coupled with supply-chain issues, led to national drug shortages and generated significant accessibility concerns for RA patients [12]. Furthermore, disruptions in routine healthcare delivery related to government-mandated lockdowns and social distancing further complicated RA disease management [11], due to limitations on in-person visits and limitations on obtaining bloodwork. Several reports have shown that many patients perceived a decline in their RA disease control because of these pandemic-related stressors, even in the absence of a COVID-19 infection [12, 13].

In effect, the sociopolitical, medical, financial, and psychological ramifications of the COVID-19 pandemic created a unique, distressing situation and opportunity to study the relationship between stress and RA disease activity in a widely affected population. Previously published reports have explored the impact of the pandemic on stress and disease flares in rheumatology patients but have focused on broad cohorts without specific emphasis on RA or provided limited detail on RA flares [11–17]. Our previous publication examined COVID-19-related stress among RA patients in the Greater Los Angeles area and demonstrated that stress negatively impacts disease activity, as measured by Routine Assessment of Patient Index Data 3 (RAPID3) [18]. However, it did not evaluate detailed metrics of flare activity. The purpose of the current study was to specifically investigate how stress influences RA flares in the context of a global pandemic through an online survey using validated measures.

## Materials and methods

### Study population

A cross-sectional survey was conducted amongst RA subjects between July 2020 and December 2020 at the University of California, Los Angeles (UCLA), with approval from the UCLA Institutional Review Board (IRB #20–000930). All subjects received care at one of ten UCLA Rheumatology clinics located in the greater Los Angeles area: Westwood, Santa Monica, Santa Clarita, Porter Ranch, Burbank, Westlake Village, Torrance, Beverly Hills, Encino, and Downtown Los Angeles. Subjects receiving invitations to complete an online survey met the following criteria: at least three electronic health record encounters with RA as coded by the International Classification of Diseases Ninth and Tenth Revision (ICD-9, ICD-10) and an active email address. All invitations included an online information sheet describing the purpose of the study and explained that their consent to participate would be provided by clicking on the survey link. The survey was conducted online over a secure REDCap tool (REDCap, Nashville, Tennessee) and originally sent to 361 subjects in July 2020 and later to 4676 more subjects in November 2020. Subjects received two reminder emails 1 week apart if they did not complete the survey after the initial invitation. Electronic informed consent was obtained at the beginning of the survey.

### Subject characteristics

Demographics including gender, race and ethnicity, body mass index (BMI), and disease duration were collected via survey as well as information on sleep quality. Age and seropositivity status (anti-cyclic citrullinated peptide antibody and rheumatoid factor laboratory values) were extracted from electronic medical records.

### RA flare and disease activity measures

Patient-reported flares were assessed using three time-oriented approaches: current flares at the time of the survey (yes/no); remote flares categorized as none, one, or several since the pandemic began (requiring patient recall); and frequency compared to pre-pandemic levels over the last 4–9 months preceding completion of the survey (decreased, about the same, increased). Current flares were also evaluated via the RA Flare Questionnaire (RA-FQ), a validated composite patient-reported outcome measure (PROM) used to identify and quantify flares. The RA-FQ examines RA disease activity using a 0–10 numeric rating scale across five items, pain, fatigue, stiffness, function, and participation, together with whether the patient is currently flaring and, if so, its duration (total score 0–50) [19].

To assess RA disease activity, subjects were asked to complete the Routine Assessment of Patient Index Data 3 (RAPID3) score [20]. The RAPID3 is a PROM containing twelve questions related to functional activity, patient pain, and patient global assessment. RAPID3 score cutoffs categorize disease activity as in near remission (score ≤ 3), low severity (4–6), moderate severity (7–12), or high severity (≥ 13) [21]. Subjects were also asked to self-report whether they were currently in remission and/or if they were in remission prior to the pandemic.

### Measures of psychological distress

The Perceived Stress Scale (PSS) is a validated self-reported measure designed to assess the “degree to which individuals appraise situations in their lives as stressful” over the last 4 weeks [22]. For this study, subjects’ stress levels were evaluated by an abbreviated, yet validated, 4-item PSS (PSS-4) [23, 24]. Subjects were also asked questions related to the COVID-19 pandemic that were adapted from previous studies, including Likert-scale questions on overall stress, apprehension, panic, helplessness, stress from work, financial stress, stress from home, and stress from social distancing measures as described in our previously published study [18, 25, 26]. These questions were found to have a Cronbach alpha of 0.88 in Zhang and Ma’s COVID-19 study and previously used in Lau et al.’s 2006 SARS epidemic study [27].

### Statistical analysis

Quantitative variables were summarized as mean ± standard deviation, and categorical variables were presented as a number or percentage. Linear regression was employed to assess the relationship between PSS-4 and other stress measures while adjusting for demographic and clinical characteristics. Predictors of flares were analyzed using three definitions: (1) current flare (presence of absence of current RA flare) modeled using logistic regression; (2) remote flares (flares occurring since the start of the pandemic) via multivariable linear regression; and (3) flare frequency compared to pre-pandemic levels (“decreased,” “about the same,” or “increased”) via logistic regression. A significance level of *p* < 0.05 was considered statistically significant.

## Results

Among 5037 RA subjects invited to participate in the study, 361 in July 2020 and 4676 in November 2020, 1138 (22.6%) completed the survey and were included in the analysis population. The demographics and clinical characteristics have been described previously [18]. The average age of respondents was 57.5 years, 79.4% were female, and 30% identified as non-white. Regarding respondent disease characteristics, 62% were seropositive (anti-cyclic citrullinated peptide antibody and/or rheumatoid factor), and 40% reported remission at the time of survey completion. Information regarding RA therapies (i.e., use of any csDMARDs, bDMARDs, and/or tsDMARDs) was analyzed and reported previously [18].

### Respondent characteristics, disease activity, and stress levels by number of RA flares

Respondents were categorized according to self-reported number of RA flares since the start of the pandemic (Table 1). Approximately 69% of respondents had a RA flare at least once, with 492 reporting several flares, 290 reporting one flare, and 346 reporting no RA flares. Those reporting no RA flares tended to be older, have lower BMI, and longer disease duration. As expected, patient global and RAPID3 increased across the groups, with subjects experiencing multiple flares scoring the highest, and those experiencing no flares reported the highest rates of remission prior to the pandemic (all *p* < 0.0001). Respondents reporting multiple flares had the highest PSS-4 scores (*p* < 0.0001) and increased stress levels overall (*p* = 0.0002). Unsurprisingly, greater than 50% of all flare groups reported increased overall stress compared to before the pandemic (no flares 57%, one flare 68%, multiple flares 72%), and less than 11% reported a decrease in stress (no flares 10%, one flare 9%, multiple flares 8%).

**Table 1:** Characteristics of respondent by number of flares since the onset of the COVID-19 pandemic

	No flares (*N* = 346)	Yes, once (*N* = 290)	Yes, several times (*N* = 492)	*p-*value
Age, mean ± SD years	60.0 ± 16.2	57.1 ± 14.3	55.7 ± 14.5	0.0003
Female, *N* (%)	255 (73.9%)	244 (84.1%)	405 (83.5%)	0.0006
Race (%)				0.0417
Caucasian	66.8%	67.4%	66.5%	
LatinX	10.6%	12.9%	17.5%	
Asian	13.2%	10.4%	7.0%	
Black	5.3%	5.2%	4.3%	
BMI, mean ± SD	25.7 ± 5.6	26.9 ± 6.2	27.5 ± 7.0	0.0004
ACPA or RF positive, *N* (%)	130 (60.5%)	140 (68.6%)	206 (58.9%)	0.0647
RA duration, mean ± SD years	16.8 ± 13.0	15.1 ± 12.0	14.6 ± 11.3	0.0312
Patient global VAS, mean ± SD, range 0–10	2.8 ± 2.4	4.3 ± 2.3	5.8 ± 2.4	< 0.0001
RAPID3, mean ± SD, range 0–30	4.3 ± 5.0	8.0 ± 5.8	12.5 ± 6.1	< 0.0001
Respondent-reported remission, *N* (%)	241 (70.7%)	128 (44.8%)	84 (17.2%)	< 0.0001
PSS-4, mean ± SD	4.7 ± 3.0	5.6 ± 3.0	6.8 ± 3.2	< 0.0001
Overall stress level (%, *N*)				0.0002
Increased	56.7%	68.3%	71.5%	
Unchanged	33.0%	23.1%	20.5%	
Decreased	10.4%	8.6%	7.9%	

*N*, number; *SD*, standard deviation; *BMI*, body mass index; *ACPA*, anti-cyclic citrullinated peptide antibody; *RF*, rheumatoid factor; *RA*, rheumatoid arthritis; *VAS*, visual analogue scale; *RAPID3*, Rapid Assessment of Patient Index Data; *PSS-4*, Perceived Stress Scale 4

### Characteristics of respondents experiencing current RA flares

Approximately 36% of respondents reported experiencing a current RA flare at the time of survey completion. They reported significantly higher patient global, RAPID3, and RA-FQ scores than those not actively experiencing a RA flare (all *p* < 0.0001) (Table 2). The current RA flare group reported a 12% remission rate prior to the onset of the pandemic, while a 57% remission rate for the no RA flare group (*p* < 0.0001). Current flare status was also associated with higher PSS-4 scores and increased overall stress levels (*p* < 0.0001 and *p* = 0.0004).

**Table 2:** Comparison of respondents by current RA flare status at time of survey

	Current flare No (*N* = 719)	Current flare Yes (*N* = 409)	*p-*value
Age, mean ± SD years	58.2 ± 15.2	56.0 ± 15.0	0.0173
Female, *N* (%)	561 (78.7%)	342 (84.4%)	0.0188
Race (%)			0.0063
Caucasian	68.4%	64.2%	
LatinX	12.2%	17.5%	
Asian	11.4%	7.2%	
Black	4.5%	5.2%	
BMI, mean ± SD	26.4 ± 6.3	27.5 ± 6.6	0.0057
ACPA or RF positive, *N* (%)	308 (65.3%)	165 (55.9%)	0.0098
RA duration, mean ± SD years	15.6 ± 12.3	14.9 ± 11.6	0.3133
Patient global VAS, mean ± SD, range 0–10	3.5 ± 2.5	6.1 ± 2.2	< 0.0001
RAPID3, mean ± SD, range 0–30	6.1 ± 5.6	13.7 ± 5.7	< 0.0001
RA-FQ score, mean ± SD, range 0–50	13.4 ± 11.4	29.7 ± 10.8	< 0.0001
Reported remission, *N* (%)	404 (57.1%)	49 (12.1%)	< 0.0001
PSS-4, mean ± SD	5.2 ± 3.1	6.9 ± 3.1	< 0.0001
Overall stress level (%)			0.0004
Increased	62.3%	72.9%	
Unchanged	26.9%	21.8%	
Decreased	10.7%	5.4%	

*N*, number; *SD*, standard deviation; *BMI*, body mass index; *ACPA*, anti-cyclic citrullinated peptide antibody; *RF*, rheumatoid factor; *RA*, rheumatoid arthritis; *VAS*, visual analogue scale; *RAPID3*, Rapid Assessment of Patient Index Data; *PSS-4*, Perceived Stress Scale 4

### RA flare frequency comparisons before and during the COVID-19 pandemic

Compared to pre-pandemic levels, 404 respondents (36.3%) reported an increase in flare frequency, while 622 (55.5%) experienced similar flare frequency, and only 94 (9.2%) reported fewer flares (Table 3). Those experiencing an increased number of flares exhibited higher patient global and RAPID3 scores, along with greater stress levels as measured by the PSS-4, and fewer reports of current remission (all *p* < 0.0001).

**Table 3:** Stratification of respondents by RA flare frequency relative to pre-pandemic levels

	Decreased *N* = 94	About the same *N* = 622	Increased *N* = 404	*p-*value
Age, mean ± SD years	55.3 ± 15.2	59.4 ± 15.3	54.5 ± 14.2	< 0.0001
Female, *N* (%)	76 (82.6%)	473 (76.4%)	345 (86.5%)	0.0003
Race, *N* (%)				0.0003
Caucasian	46 (49.5%)	424 (69.3%)	268 (67.0%)	
LatinX	25 (26.9%)	68 (11.1%)	63 (15.8%)	
Asian	12 (12.9%)	70 (11.4%)	27 (6.8%)	
Black	6 (6.5%)	28 (4.6%)	20 (5.0%)	
BMI, mean ± SD	26.9 ± 6.7	26.6 ± 6.2	27.1 ± 6.6	0.45
ACPA or RF positive, *N* (%)	48 (70.6%)	256 (62.8%)	167 (58.4%)	0.15
RA duration, mean ± SD years	12.9 ± 11.8	15.9 ± 12.5	15.3 ± 11.7	0.085
Patient global VAS, mean ± SD	3.5 ± 2.7	3.8 ± 2.5	5.9 ± 2.3	< 0.0001
RAPID3, mean ± SD, range 0–30	5.9 ± 5.6	6.7 ± 6.0	12.9 ± 6.0	< 0.0001
Current remission, *N* (%)	52 (56.5%)	324 (53.0%)	71 (17.7%)	< 0.0001
PSS-4, mean ± SD, range 0–40	5.6 ± 2.9	5.0 ± 3.1	7.2 ± 3.1	< 0.0001

*N*, number; *SD*, standard deviation; *BMI*, body mass index; *ACPA*, anti-cyclic citrullinated peptide antibody; *RF*, rheumatoid factor; *RA*, rheumatoid arthritis; *VAS*, visual analogue scale; *RAPID3*, Rapid Assessment of Patient Index Data; *PSS-4*, Perceived Stress Scale 4

### Regression models for RA flare status, number of occurrences, and frequency

Regression modelling demonstrated that PSS-4 score, financial stress, and sleep quality were all significantly associated with current flare (all *p* < 0.03) (Table 4). The number of remote flares since the pandemic onset was found to positively correlate with PSS-4, financial stress, and home stress (all *p* < 0.03). PSS-4, feelings of apprehension and panic, financial stress, and sleep quality were associated with RA flare frequency (all *p* < 0.05). In contrast, Asian race negatively correlated with the number of remote flares since pandemic onset as well as RA flare frequency (*p* = 0.05 and *p* = 0.02).

**Table 4:** Logistic regression analyses examining RA flare status, number, and changes in frequency relative to demographics and stressors

	Current RA flare status (Y/N)		RA flares since pandemic onset (Y/N)*		RA flare frequency compared to pre-pandemic levels**	
	Odds ratio (95% CI)	*p-*value	Odds ratio (95% CI)	*p-*value	Odds ratio (95% CI)	*p-*value
Age	1.00 (0.99, 1.01)	1.00	0.99 (0.98, 1.00)	0.14	0.99 (0.98, 1.00)	0.23
Female	1.04 (0.69, 1.57)	0.85	1.40 (0.95, 2.08)	0.10	1.11 (0.77, 1.60)	0.57
BMI	1.01 (0.99, 1.03)	0.40	1.02 (1.00, 1.05)	0.10	0.99 (0.97, 1.02)	0.55
Disease duration	0.99 (0.98, 1.01)	0.32	0.99 (0.98, 1.01)	0.29	1.01 (1.00, 1.02)	0.25
Race	-	-	-	-	-	-
LatinX	1.30 (0.84, 2.02)	0.72	0.95 (0.58, 1.55)	0.96	0.76 (0.51, 1.14)	0.25
Asian	0.85 (0.50, 1.44)	0.03	0.59 (0.35, 0.99)	0.05	0.58 (0.37, 0.93)	0.02
Black	1.74 (0.82, 3.68)	0.48	0.80 (0.36, 1.78)	0.63	0.99 (0.46, 1.81)	0.90
Other	2.77 (1.33, 5.18)	0.03	1.63 (0.70, 3.82)	0.12	1.82 (0.92, 3.60)	0.02
Seropositivity (ACPA and/or RF)	1.06 (0.94, 1.20)	0.37	0.85 (0.75, 0.97)	0.02	0.99 (0.89, 1.10)	0.83
PSS-4	1.13 (1.07, 1.19)	< 0.0001	1.11 (1.05, 1.17)	0.0004	1.07 (1.02, 1.13)	0.01
Apprehension	-	-	-	-	1.27 (1.02, 1.59)	0.04
Panic	-	-	-	-	1.49 (1.18, 1.89)	0.001
Financial stress	1.40 (1.16, 1.69)	0.0004	1.35 (1.10, 1.66)	0.004	1.37 (1.15, 1.62)	0.0004
Home stress	-	-	1.29 (1.04, 1.59)	0.02	-	-
Sleep quality	1.20 (1.03, 1.41)	0.02	-	-	1.20 (1.03, 1.38)	0.02

^*^Remote flares—number of flares since pandemic onset categorized as none versus 1 or more

^**^Relative flare frequencies—comparison of pandemic to pre-pandemic levels—categorized as much decreased, decreased, similar, increased, or much increased

*RA*, rheumatoid arthritis; *Y/N*, yes or no; *CI*, confidence interval; *BMI*, body mass index; *ACPA*, anti-cyclic citrullinated peptide antibod; *RF*, rheumatoid factor; *PSS-4*, Perceived Stress Scale 4

## Discussion

Understanding how stress exacerbates RA disease activity is essential for effective management as flares complicate treatment strategies and impair patient quality of life [28]. However, very few studies have been able to explore this relationship at the population level, particularly in the context of large-scale stressors given the rarity of such events. The COVID-19 pandemic introduced additional challenges for RA patients, escalating stress and further complicating RA management, thus providing a valuable opportunity to study RA-stress dynamics. This large, cross-sectional study confirmed that most respondents experienced greater-than-usual levels of stress during the COVID-19 pandemic. Respondents who experienced flares during the pandemic were predominantly those who were not in remission prior to its onset, suggesting RA patients with active disease are more susceptible to stress-related flares. Furthermore, the number of reported RA flares, current flare, and RA flare frequency was associated with higher stress levels, as measured by PSS-4, as well as financial stress. One unanticipated demographic finding was that individuals identifying as of Asian descent experienced fewer flares and at a lower frequency compared to other racial groups. Given the scarcity of research in this area, this study delivers valuable insights into the complex interplay between stress and RA amid a historically unprecedented global event, making it an important addition to the literature.

RA flares have drawn increasing literary attention in recent years; however, a pre-pandemic baseline flare rate for comparison has not been established. In this study, flares were assessed using three patient-reported, time-oriented measures with 36% reporting a current flare, 69% having had at least one flare in the preceding months, and 36.3% perceiving an increase in flare frequency compared to pre-pandemic levels. However, due to methodological differences, direct numerical comparisons with other studies are limited. An international survey by Myasoedova et al. using the FLARE-RA tool similarly assessed patient-reported flares in 474 patients and reported current flare rates ranging from 26 to 47% across four countries, with the US cohort reporting the lowest at 26%, lower than our 36% [29]. Additionally, Raheel et al.’s single-center retrospective review of 650 RA patients in the USA determined a 17% visit flare rate using the OMERACT 9 flare definition (worsening disease activity prompting the initiation, change, or increase of therapy) [30]. Overall, our flare rates appear elevated (but interpreted with caution), and the patient-perceived increase in pandemic flare frequency represents a unique patient-reported outcome new to the literature, calling further attention to the importance of stress-RA flare dynamics.

A major finding of clinical relevance from this study is that pandemic-related stress is associated with increased RA flares. While our cross-sectional survey cannot establish causality, expanding evidence supports a biological link between psychological stress and RA disease activity [31]. RA patients exhibit dysfunction in their stress response systems mediated by the hypothalamic–pituitary–adrenal (HPA) axis and sympathetic nervous system (SNS), where stress intensifies inflammatory load, increasing pro-inflammatory cytokines implicated in RA pathogenesis (i.e., IL-1β, IL-6, and TNF-α) [32–34]. In Straub et al.’s “two-hit model” of stress-induced inflammation, RA patients with genetic susceptibilities in inflammatory pathways and/or HPA axis-SNS desynchronization sustain chronic immune activation (first hit) and when exposed to stress, especially chronic, akin to that during the COVID-19 pandemic, mount a more robust proinflammatory response (second hit) [35]. Literature suggests that the stress-activated NOD-like receptor protein 3 (NLRP3) inflammasome may be an interface between stress, depression (a common RA comorbidity), and systemic inflammatory diseases like RA by upregulating IL-1β [36]. Interestingly, the NLRP3 inflammasome has also been implicated in RA, and mRNA viruses like SARS-CoV-2 have been shown to be stimulatory, potentially driving persistent inflammation in post-viral syndromes like long COVID [37–40]. However, in our cohort, only 3.4% of respondents reported a confirmed COVID-19 self-infection, 2.6% household member infection, and 41.7% non-household infection, pointing to limited direct viral exposure and suggesting that psychological stress, rather than infection, was a more likely contributor to flares in our findings [18]. From a therapeutic perspective, stress has been shown to limit the response to DMARDs, like methotrexate, in preclinical mouse models, and worse mental health scores were independently associated with risk of flare when tapering RA patients off TNF-α inhibitors in the OPTTIRA trial [41, 42]. These studies highlight the broader impact of stress on RA, demonstrating it not only exacerbates RA disease activity but also thwarts management.

The present study’s findings have several important implications for the management of RA. The most compelling finding, the self-reported increase in the number and frequency of RA flares during the COVID-19 pandemic, underscores the need for clinicians and patients to consider changes in management and treatment strategies during periods of heightened stress. These situational adjustments may include more frequent disease activity monitoring, potential modifications to DMARD therapy, and an emphasis on adjunctive stress management techniques to prevent and treat stress-related flares. It is important to note that this cross-sectional survey was not designed to capture detailed or time-sensitive data on clinical disruptors such as medication adherence, DMARD adjustments, or access to rheumatologic care that would allow for inclusion as confounders in regression analyses and enable causal inferences with RA flares. In our previous report of the same cohort, 3% of participants discontinued RA medications under medical guidance, another 3% on their own, and 11.5% reported difficulty obtaining their medications for reasons related to COVID-19 [18]. Furthermore, 62.4% switched to telehealth appointments, 8.4% had their rheumatologist cancel a clinic visit, and 15.6% cancelled clinic appointments themselves. Limited studies have touched on these variables in the context of the early pandemic, but further study is needed to clarify their temporal and causal relationship with RA flares [11, 12, 14, 16].

Another clinically relevant finding was the vulnerability of respondents not in remission before the pandemic, as they exhibited a greater likelihood of experiencing flares than those with better-controlled disease activity. This suggests clinicians should be particularly vigilant in identifying RA flares in this population during stressful events. Enhanced monitoring and proactive management as noted above may be of value to prevent RA exacerbations in this group. In addition, the negative association between Asian race and RA flares suggests there may be knowledge to be acquired in understanding how this patient population perceives and manages RA flares during times of heightened stress that can be applied to other racial/ethnic groups. Overall, these results reinforce the need for a comprehensive RA management approach that addresses psychological stressors and individual vulnerabilities to flare. The three most significant stressors found to be associated with flares—financial stress, home stress, and poor sleep—should not be overlooked in care plans.

Other studies conducted during the COVID-19 pandemic have explored the impact of stress on disease activity and reported similar findings. From a broader perspective, Iannuccelli et al. conducted an online survey with a cohort of 194 patients at the University Hospital of Rome, Italy (82 with RA, 72 with fibromyalgia (FM), and 40 healthy controls (HC)), to assess the impact of lockdown measures on the emotional wellbeing and disease activity of RA and FM patients [17]. Their results revealed the pandemic may have had significant psychological consequences for RA respondents, with 14.6% and 17.1% experiencing anxiety and depression worthy of psychiatric attention (assessed via the Zung Anxiety and Depression Self-Assessment Scales, respectively). RA respondents demonstrated greater emotional resilience compared to those with FM and HCs on a 14-item resiliency scale (*p* < 0.05). However, those with RA demonstrated a significant worsening in disease activity, as measured by the RA Disease Activity Index-5 (RADAI-5), when compared to historic pre-pandemic levels, even though there were no changes in pain levels in the post-lockdown cohort. When evaluating the findings of the current study alongside those of Iannuccelli et al., both highlight the pandemic’s negative psychological impact on RA patients, as well as its exacerbation of disease activity. Importantly, this study offers a more targeted investigation of RA, with a particular focus on RA flares utilizing a larger RA cohort.

In another study, Maldonado et al. surveyed 1692 adult and pediatric rheumatology patients during the pandemic’s peak in the Bronx, New York, noting 41% reported flares, and high levels of COVID-related distress were associated with worsened disease activity and increased odds of flares [16]. Notably, 16% faced medication access issues, which were associated with a fourfold increase in flare likelihood, compared to 11.5% in our population as reported earlier [18]. Their findings also demonstrate a significant increase in disease activity during the pandemic. However, Maldonado et al.’s study did not specifically focus on RA and had limitations in its flare assessments, only asking if respondents had a “flare in their rheumatologic condition” and the intensity (mild, moderate, severe) without validated outcome measures [16], compared to our detailed evaluation of three outcome metrics describing RA flares. In addition, Maldonado et al. describe that systemic lupus erythematosus patients were largely impacted by stress. Furthermore, this study examined how additional stressors, such as homelife, finances, and sleep, affect disease activity and flares, adding more detail to the picture.

This study has several strengths that add to existing literature on stress and RA. The large size, relatively high response rate, and diversity of the cohort increase the applicability of its findings across various populations. Its specific focus on RA, rather than multiple rheumatologic diseases, allowed for more targeted questions for a thorough examination of flare patterns. Unlike prior studies that assessed flares in a limited fashion (i.e., flare occurrence with a simple yes/no evaluation), this study employed flare-specific questions to capture more detailed metrics, including current flare status, total number of flares since the pandemic, and compared perceived flare frequency to pre-pandemic levels to capture a more comprehensive picture of flare patterns. Furthermore, this study utilized validated disease activity measures, the RA-FQ and RAPID3, to ensure standardized assessment of disease activity, as well as the PSS-4 and COVID-19 pandemic Likert questions to investigate the associations between stress and RA flares.

On the other hand, several potential limitations need to be acknowledged. Firstly, this study was conducted at a single academic center, which may limit its generalizability. However, our findings correspond with international studies, such as the RA Narrative Study that spanned five countries: Brazil, Canada, France, Japan, and the USA [14]. Secondly, the study survey was administered in July and November of 2020, prior to January 2021, the peak of COVID-19 cases in Los Angeles. Another potential limitation of this study is the survey response rate of 22.6%, which raises concerns of possible selection bias. Non-respondents may differ systematically from respondents, such as higher stress levels, disease severity, or limited digital access, which could impact generalizability, especially among vulnerable populations. Additionally, self-selection bias may have influenced participation, with individuals experiencing higher disease burden or stress more motivated to respond. While appearing low, this response rate is higher than other RA studies conducted during the COVID-19 pandemic [11, 16]. A recent analysis of five major US national and state surveys found an average 29% relative reduction in response rates following the onset of the pandemic with the greatest declines in response and coverage occurring among lower-income, less-educated, and racially minoritized groups [43]. In our previous report of this cohort, we found no differences in age, gender, or BMI between respondents and non-respondents, though a higher proportion of whites completed the survey compared to non-whites (26.6% vs 20.3%, *p* < 0.0001) [18]. As such, this study’s findings require interpretation within the unique sociopolitical context of the pandemic and highlight the need for more inclusive methodologies that address complex digital and cultural barriers during public health crises. Like many self-reported surveys, this study is not exempt from subjectivity, recall biases, and was only available in English. On the other hand, surveys carry potential to capture patient perspectives, which is of value when assessing stress and flares. Regarding stress measurements, the PSS-4 is not specific to pandemic stressors; however, several pandemic-specific Likert questions were administered as part of the survey. Furthermore, a cross-sectional study cannot evaluate the “temporality” of either the stress or flare measures. Finally, the study cannot establish a definitive causal relationship as it is possible respondents experiencing flares may be more likely to experience and report stress when compared to non-flaring respondents.

To build upon these findings, conducting prospective longitudinal studies with detailed documentation of stressors and medication usage would be ideal, though challenging to execute. Inclusion of in-person assessments, such as the Clinical Disease Activity Index (CDAI) or Disease Activity Score 28 (DAS28), would provide physician evaluations and complement patient-reported outcomes. A closer examination of racial differences in stressors and flare patterns could offer more details on demographic variations and their impact on RA management. Additionally, broadening the study to include multiple languages in addition to English would improve its inclusivity and accommodate a wider range of patient experiences and perspectives.

In conclusion, this study demonstrates increased stress may significantly contribute to both a higher number and frequency of RA flares. These findings support the incorporation of stress management into RA treatment strategies with close attention to stressors related to finances, household matters, and sleep. Future investigations are required to further explore these relationships and develop comprehensive approaches that support RA patients in managing both their rheumatologic disease and stress to achieve more favorable health outcomes.

## Data Availability

N/A.

## Code availability

N/A.
